# Operation-specific and time-resolved monitoring of occupational nano/sub-micron particle exposure in a Swedish metal additive manufacturing facility

**DOI:** 10.1093/annweh/wxag040

**Published:** 2026-06-01

**Authors:** Lena Andersson, Andi Alijagic, Anders Johansson, Magnus Engwall, Eva Särndahl, Alexander Hedbrant

**Affiliations:** Department of Occupational and Environmental Medicine, Faculty of Medicine and Health, Örebro University, Örebro SE-701 82, Sweden; Inflammatory Response and Infection Susceptibility Centre (iRiSC), Faculty of Medicine and Health, Örebro University, Örebro SE-701 82, Sweden; Inflammatory Response and Infection Susceptibility Centre (iRiSC), Faculty of Medicine and Health, Örebro University, Örebro SE-701 82, Sweden; School of Medical Sciences, Faculty of Medicine and Health, Örebro University, Örebro SE-701 82, Sweden; Man-Technology-Environment Research Centre (MTM), Faculty of Business, Science and Engineering, Örebro University, Örebro SE-701 82, Sweden; Department of Occupational and Environmental Medicine, Örebro University Hospital, Örebro SE-701 85, Sweden; Man-Technology-Environment Research Centre (MTM), Faculty of Business, Science and Engineering, Örebro University, Örebro SE-701 82, Sweden; Inflammatory Response and Infection Susceptibility Centre (iRiSC), Faculty of Medicine and Health, Örebro University, Örebro SE-701 82, Sweden; School of Medical Sciences, Faculty of Medicine and Health, Örebro University, Örebro SE-701 82, Sweden; Inflammatory Response and Infection Susceptibility Centre (iRiSC), Faculty of Medicine and Health, Örebro University, Örebro SE-701 82, Sweden; School of Medical Sciences, Faculty of Medicine and Health, Örebro University, Örebro SE-701 82, Sweden

**Keywords:** 3D printing, laser powder bed fusion, emission, dust exposure, post-processing, nanoparticles

## Abstract

The aim of the study was to determine nano/sub-micron particle and dust exposure levels throughout the whole workflow at a Swedish metal additive manufacturing (AM) facility, focusing on the laser powder bed fusion (L-PBF) method. By evaluating particle levels and composition across different AM processes using both stationary and personal sampling, the study sought to improve exposure assessment and inform protective measures in the metal AM workplaces. Measurements were conducted during five measurement weeks, as five working days Monday–Friday, between October 2020 and October 2023. Personal particle measurements in the breathing zone were performed on Mondays and Fridays for 1 to 3 workers per day. Stationary particle and dust sampling were performed continuously at three locations each week to capture task-specific and temporal variation in emissions. Nano/sub-micron particle concentrations ranged from 0 to 3.3 million particles/cm^3^, with the highest peaks recorded in the post-processing area. Elevated levels were also detected, near the depowdering machine, by the bandsaw, and in the lunchroom, while levels near the printers were low (<10,000 particles/cm^3^). Personal exposure peaks occurred during printer cleaning, feedstock powder filling, dust removal with compressed air, post-processing, and packing. In contrast to the increased nano/sub-micron particle levels observed, respirable, and inhalable dust levels were very low. The study highlights the need to monitor particle exposure during both manufacturing and post-processing. Health risks associated with airborne particles are influenced by both exposure levels and the toxicological properties of materials. To ensure a safe and sustainable future for metal AM, comprehensive exposure assessment, risk evaluation, and the implementation of protective measures remain essential.

What’s important about this paper? This study assessed exposures to nano- and sub-micrometer-sized particles in a metal additive manufacturing facility that utilizes laser powder bed fusion methods. Peak exposures occurred primarily during post-processing of printed components, but emissions occurred consistently from enclosed printers. Exposure monitoring and control strategies must address both manufacturing and post-processing tasks in additive manufacturing.

## Introduction

Additive manufacturing (AM), also known as industrial 3D printing, has rapidly advanced over the past decade (Gokuldoss et al. 2017; Kumar et al. 2021), and revolutionized manufacturing by enabling the creation of solid objects with virtually every geometry using computer-aided design (CAD) models (Srivastava and Rathee 2022). Compared to traditional manufacturing, AM offers several advantages, including design versatility, rapid prototyping, customization, and reduced material waste (Javaid et al. 2021). In recent years, metal AM has gained significant traction, enabling the fabrication of highly complex metal components with improved mechanical properties (Kumar et al. 2021). Its applications span diverse fields, from biomedicine (eg, patient-specific implants) to the aerospace industry (eg, turbine blades for jet engines). Since AM involves diverse printing methods with different processes and feedstock materials (Herzog et al. 2016), distinct emission profiles and occupational exposure risks will be evident during the processes.

Laser powder bed fusion (L-PBF) is among the most important production categories in the metal AM industry due to its high precision, material efficiency, and ability to produce complex geometries with minimal waste (King et al. 2015). L-PBF commonly utilizes gas-atomized micron-sized powders (typically ranging from 10 to 100 µm) as feedstock for fabricating various components (Singh et al. 2017), but the particle size distribution of these powders varies depending on the material, often containing coarse (2.5 to 10 µm), fine (<2.5 µm), and ultrafine (<100 nm) particles (Anderson et al. 2018; Alijagic et al. 2024). The presence of fine particles in virgin powder generally increases the powder density, which in turn reduces surface roughness and minimizes defects of the printed parts (Muñiz-Lerma et al. 2018). In L-PBF, the printer is loaded with feedstock powder composed of various types of metal alloys tailored to the intended product. A laser beam selectively melts the metal layer by layer, allowing the material to condense and form the desired structure (King et al. 2015). During the L-PBF processes, emission of fine and ultrafine particles (nanoparticles) may be released, contributing to occupational exposure, particularly during powder handling, sieving, cleaning, printer refilling, and post-processing of printed components (Taylor et al. 2021). Particulate materials used in AM are not only present in powder feedstocks but can also be unintentionally generated during the printing process itself (Taylor et al. 2021), including particles of various sizes, from nanoparticles to larger aggregates/agglomerates (Vallabani et al. 2022; Alijagic et al. 2023). As a result, inhalation and dermal exposure to both feedstock particles and unintentionally formed particulate byproducts may occur throughout the entire AM workflow (Alijagic et al. 2022). Fine and ultrafine particles can easily become aerosolized, with inhalable (<100 µm), thoracic (<10 µm), and respirable (<4 µm) fractions posing potential health risks during various L-PBF operations (Brown et al. 2013). Additionally, metals used in L-PBF techniques, such as nickel, cobalt, or chromium, may have toxic or sensitizing properties (Misra et al. 2012; Bonefeld et al. 2015; Buters and Biedermann 2017). In addition, changes in the physicochemical properties of feedstock particles during printing, such as size, shape, porosity, surface topography, interfacial free energy, chemical composition, and surface oxidation, can significantly influence biochemical mechanisms when particles interact with biological systems upon inhalation or dermal exposure (Rahmati et al. 2020). This aspect of particle safety assessment is crucial for classifying materials based on their toxic potential. For example, the reuse of powder in L-PBF may alter its physicochemical properties and lead to changes in elemental composition compared to virgin powders, which could impact occupational exposure and toxicity (Slotwinski et al. 2014; Alijagic et al. 2022). Furthermore, discussions on risk assessment and management have emphasized the urgent need for improved exposure characterization in AM occupational settings (Assenhöj et al. 2023). While metal exposure has been linked to adverse effects on multiple organs, including the respiratory tract (Nemery 1990), the kidneys (Kim et al. 2015), liver (Boey and Ho, 2020), and cardiovascular system (Pan et al. 2024), the specific health implications of particles generated during AM processes remain insufficiently explored.

A key challenge in addressing these concerns is the limited data on occupational exposure in metal AM, largely due to the absence of standardized methods for particle sampling — especially for nanoparticles, which pose the greatest concern. While some measurements of nano- or sub-micron-sized particles have been conducted in the AM industry, primarily using logging instruments that detect particle concentrations in the air (Runström Eden et al. 2022; Assenhöj et al. 2023), knowledge about these particles, their contribution to emissions across different AM operations, personal exposure levels, and variations over time remains insufficient. Nano/sub-micron-sized particles remain airborne for extended periods due to their small size, low density, and slow settling rates, increasing the likelihood of inhalation, particularly in settings where ventilation systems may not effectively remove them. These particles can stay suspended for several hours, with deposition rates decreasing as humidity rises (Wang et al. 2017). The knowledge gap on the toxicity of AM-generated nanoparticles is concerning, especially since AM is recognized as a Key Enabling Technology by the EU (STOA, 2021), expected to transform global markets and drive industrial innovation. Addressing these gaps is crucial for ensuring the safe and sustainable advancement of AM technologies while mitigating potential occupational and environmental health risks through the implementation of effective protective measures in AM workplaces.

The primary objective of this study was to evaluate the feasibility of measuring as well as collecting nano/sub-micron-sized particles in a Swedish metal AM facility, with a specific focus set on the L-PBF printing method. By assessing particle levels and composition across different AM operations using both stationary and personal measurements, this study aimed to enhance exposure assessment. The findings will contribute to advancing research on potential health effects and to inform the development of effective protective measures in AM workplaces.

## Methods

### Description of the additive manufacturing facility

The study was performed at a Swedish AM company from 2020 to 2023. The company had five 3D printers, of which four were metal printers, including two EOS M 290 printers, one for Ni/Fe printing and one for Ti printing, one SLM 500 for Al printing, and an Aconity Midi printer that was used as an experimental printer. One printer (Ultimaker) was used for polymeric materials (polyamide-based feedstock powder). The products could be produced in sizes up to Ø 170 mm × H 200 mm (Aconity Midi printer), 250 mm × 250 mm × 325 mm (EOS M 290 printer), and 500 mm × 280 mm × 365 mm (SLM 500 printer). The printing time for these products ranged from a few hours to 100 hours. The post-processing room contained a bench for manual post-processing and two blast machines (DryBlast RS Challenger1000 and HGH 6,040). Outside the post-processing room, in the area for packaging of printed parts, there were three post-processing machines: a depowdering machine (Sulokon SMF-AT300), heat treatment machine (Nabertherm), and a band saw (Klaeger VSB 500 3D Cut). The ventilation consisted of mechanical supply and exhaust air. In addition to general ventilation, there was extra exhaust ventilation in the printers and post-processing machines as well as a movable point exhaust ventilation in the post-processing room.

There were five workers who handled the printers and post-processing. The number of employees increased during the study period, from 24 in 2020 to 28 in 2023. In addition to printer operators, employees included office workers and staff working in the material laboratory. The worktime was daytime.

### Description of production activities

The production of 3D-printed parts varied depending on the assignment. The company had serial production and worked with projects to produce prototypes. The time to print an object depends on the size and varied between a few hours to 100 hours. The printers worked largely unmanned during printing, with cleaning, removal of printed parts, and refilling of powder taking place between prints. The printer SLM 500 with 4 laser zones took 24 hours for a large build, but normally they had 12 to 24 hours of printer time per printed part. For the printer EOS M 290, a 10 to 15 cm print took 100 hours with one refill of powder, but normally they printed smaller parts that took 24 to 48 hours to complete. The feedstock powder alloys and their chemical composition can be found in Table S1.

### Description of worker protection measures

The workers used full protective equipment with a filter-fed fresh air ventilator, overall and gloves when doing specific work tasks at the printers, such as changing of printed parts, cleaning, and powder filling, but not for surveillance. From the start of the project in 2020 until August 2023, no personal protective equipment (PPE) was used in the post-processing room. From August 2023, the workers used the same protective equipment for work in post-processing as for work by the printers. They used the Sundström filter fan SR 500 with mask SR 570 or the 3M™ Versaflo fan system. Both systems have the protection level TH3 and filter class P3 in the masks.

### Measurement and sampling points

Measurements were made in three different buildings: lab/production, office, and CNC (Computer Numerical Control) building. Most measurements, both personal and stationary, took place in the lab/production building. The lab/production building included all five printers, each placed in a separate room, a post-processing room, an area for packaging of printed parts, a laboratory for sample preparation, tensile/hardness/impact testing, and microscopy, offices, conference rooms, and a small lunch room.

The CNC building had the CNC machine for grinding and cleaning printer plates to be reused, and an office. A single personal measurement was performed on a CNC operator in the CNC building. The office was shared with another company and included three conference rooms, an office landscape, and a large lunch room.

### Measurement design and implementation

Measurements were performed during five measurement weeks as five working days Monday–Friday: 19 to 23 October 2020, 11 to 15 October 2021, 14 to 18 March 2022, 10 to 14 October 2022 and 16 to 20 October 2023. Personal measurements were only performed by logging of air particles with a size of 10 to 300 nm in the breathing zone, on Mondays and Fridays during the five measurement weeks, on 1 to 3 workers/day. Stationary measurements were performed both by logging air particle measurements (10 to 300 nm) and by stationary pumped sampling of inhalable dust, respirable dust, total dust, PM2.5, PM10, and particle sampling by a cascade impactor were performed throughout the working week, from Monday morning to Friday afternoon at three sampling places per measurement week (for technical details see *Measurement equipment* below). The sampling time was around 100 hours, with the exception of two cascade impactor measurements where shorter sampling times of 37 to 50 hours were performed because the pumps stopped running. Measurements of temperature and relative humidity were also performed at the stationary sampling places. For stationary sampling, a tripod or a small cart with sampling equipment was placed as close to the emission source as possible without disrupting regular work, ie 1 to 2 m from the emission source and approximately 1 to 1.5 m above the ground.

For occupational dust exposure measurements, the normal procedure is to measure the exposure during a working day, or part of a working day, for comparison with occupational exposure limits (OEL) (Swedish Standard 1993, 2018; SWEA, 2023). The prolonged sampling time of a whole working week for dust, from Monday afternoon to Friday morning, was decided after initial test measurements of respirable and inhalable dust that all showed results below the laboratory detection limit. For this reason, filter-based sampling of dust was only performed by stationary sampling.

Sampling places were chosen in consultation with the company management and representatives for the project. In total, 15 area exposure measurements at 8 different sampling locations and 26 personal air particle measurements (10 to 300 nm) on 12 different workers were performed. The personal and stationary sampling, presented in Table 1, were chosen to cover the tasks and places where exposure to metal nanoparticles may occur, and places with background levels, eg, offices and lunch room. No measurements were performed by the polymer printer since it was not used as frequently as the metal printers by the company.

**Table 1:** Number of personal measurements/working tasks and number of stationary measurements/sampling place or work task for measurements at an additive manufacturing facility during the five measurement weeks.

Number of personal measurements	Number of stationary measurements	Measurement place/working task	Machine	Location/ building
5	…	Office	…	Lab building
1	…	Office	…	Office
20	…	Production	…	Lab building
…	4	Post-processing (PP)	…	Lab building
…	3	Ni/Fe printer	EOS M 290	Lab building
…	1	Al printer	SLM 500	Lab building
…	1	Experiment printer	Aconity Midi	Lab building
…	3	Lunch room	…	Office
…	1	CNC machine	Emco maxmill 630	CNC
…	1	PP—Depowdering machine	Sulocon, SMF-AT300	Lab building
…	1	PP—Band saw	Klaeger VBS 500 3D Cut	Lab building

### Measurement equipment

Personal and stationary particle measurements were performed with the NanoTracer XP (Oxility BV, Venray, The Netherlands) or the Partector 2 (Naneos, Windisch, Switzerland) measurement devices, both measuring particles in the size range of 10 to 300 nm, in the concentration range between 0 and 10^6^ particles/cm^3^. Given that nanoparticles are defined as particles <100 nm, we have in this paper chosen to name the 10 to 300 nm particles detected by these instruments as nano/sub-micron sized particles. The NanoTracer was set on a log interval of 10–16 seconds and the Partector on a log interval of 1 second. Data from a parallel measurement using both devices found the performance of the instruments to be comparable. A few peak values displayed higher particle concentrations with the Partector, possibly due to the different logging intervals, where very transient peaks could be captured by the more frequent log interval of the Partector (Fig. S1).

Stationary measurements of inhalable dust were performed with a CIS sampler equipped with a 37 mm Ø cellulose filter, 3 µm pore size. Stationary measurement of respirable dust was performed with a SKC aluminum cyclone (SKC, Eighty Four, PA, USA) connected to a filter cassette with a 25 mm Ø cellulose filter, 3 µm pore size. The CIS sampler and the filter cassette were connected to an air sampling pump (SKC Aircheck XR5000) with an air flow of 3.5 L/min for inhalable sampling and 2.5 L/min for respirable sampling. Total dust was sampled with an open face cassette on a 25 mm Ø cellulose filter, 3 µm pore size, and an air flow of 2.0 L/min. Sampling of PM 2.5 and PM10 was performed with aluminum cyclones (ChemPass^TM^ Pumping System, Ruprecht & Patashnick Co., Inc, Albany, NY, USA) with a 37 mm Ø nitrocellulose filter, 0,8 µm pore size, connected to an air sampling pump (SKC Aircheck XR5000) with an air flow of 1.8 and 4.0 L/min, respectively.

The Sioutas Cascade Impactor (SKC) was connected to an SKC Leland Legacy air sampling pump (SKC) with the air flow of 7.0 to 8.6 L/min. The impactor separates and collects airborne particles in five size ranges: >2.5 µm, 1.0 to 2.5 µm, 0.50 to 1.0 µm, 0.25 to 0.50 µm, and <0.25 µm. The impactor was mounted with four 25 mm Ø, 3 µm pore size, MCE membrane filters and one 37 mm Ø, 5 µm pore size MCE membrane filter.

Workers with personal sampling kept a work diary on Monday and Friday and were in addition interviewed to retain information on the work tasks they performed during the measurements that could be connected to the logging instruments afterwards. Temperature and relative humidity during the measurement weeks were logged every 5 minutes at each stationary measurement place by Testo 174H (Nordtec, Gothenburg, Sweden).

### Analytical methods for dust and metal exposure assessment

Analyses of dust and metals in dust were performed by the laboratory at the Department of Occupational and Environmental Medicine, Örebro University Hospital, Sweden. The laboratory is accredited by Sweden's national accreditation body SWEDAC (Certificate No 1351). Analyses of inhalable dust, respirable dust, total dust, PM 2.5, PM 10, and the filters from the Sioutas Cascade Impactor were performed gravimetrically. The measured dust mass on the filters was recalculated to mg/m^3^. Metals in the filters for inhalable dust, respirable dust total dust, and from the Sioutas Cascade Impactor were determined by dissolving the filters in concentrated nitric acid and 10% hydrogen peroxide and analyzed with inductively coupled plasma mass spectrometry (ICP-MS) with an iCAP Q ICP-MS instrument (Thermo Fisher Scientific, Waltham, MA, USA). The metals analyzed included Al, As, Ba, Be, Ca, Cd, Co, Cr, Cu, Fe, Mg, Mn, Mo, Ni, Pb, Sb, Tl, V, and Zn. The limit of quantification (LoQ) was 0.100 mg/sample for gravimetrical analysis of dust and for metals, the LoQ varied between 0.010 to 20 µg/sample (0.010 µg/sample for Tl; 0.030 µg/sample for Pb; 0.10 µg/sample for Be, Cd, Co, Cr, Cu, Mn, Mo, Sb and V; 0.20 µg/sample for As, Ba, Ni and Zn; 2.0 µg/sample for Fe and Mg; 20 µg/sample for Al and Ca).

### Statistical analysis

Standard parameters (AM, SD, GM, GSD, range) were calculated for the measurements of nano/sub-micron particles (10 to 300 nm), inhalable dust, respirable dust, total dust, PM 2.5, PM 10, the samples from the Sioutas Cascade Impactor, and metals in dust. For the analytical limit of detection (LoD), a signal-to-noise ratio of 3 was used, defined as the concentration of 3 standard deviations of the blank (Welac EuroChem 1993). For the laboratory analytical LoQ, a signal-to-noise ratio of 10 was used, defined as the concentration of 10 standard deviations of the blank.

## Results

### Stationary nano/sub-micron particle monitoring across the additive manufacturing facility

In order to understand the spatial and temporal distribution of nano/sub-micron particles during different AM operations, stationary logging measurements of particles (10 to 300 nm) were performed from Monday afternoon to Friday morning. The occurrence of particles varied depending on locations and operations, ranging from 0 to 3,254,356 particles/cm^3^, with the highest peak measured in the post-processing area (Table 2). When comparing the highest peak of particles for each measurement location, post-processing demonstrated the highest peak values compared to all other locations. The location with the second highest peak value was the lunch room (62,062 particles/cm^3^), followed by the de-powdering machine (39,208 particles/cm^3^), band saw (30,021 particles/cm^3^), and the lowest detected levels were found at the location of the different printers (< 10,000 particles/cm^3^). When comparing the average levels over a work week, the highest levels were found in the post-processing area (GM: 2,357 ± 696 particles/cm^3^, *n* = 4) and by the depowdering machine (GM: 2,992 particles/cm^3^, *n* = 1). All other areas (printers, band saw, lunch room) detected similar average nano/sub-micron particle number (GM: 1,356 ± 260 particles/cm^3^, *n* = 8).

**Table 2:** Number of nano/sub-micron (10 to 300 nm) particles/cm^3^ by stationary measurements with NanoTracer and Partector during the five measurement weeks, Monday-Friday, at a Swedish AM company, 2020 to 2023, as presented by measurement place.

Measurement place	Measurement week	AM	Median	SD	GM	GSD	Min	Max
					(particles/cm^3^)			
Post-processing	19 to 23 Oct 2020	1,894	1,907	0.54	1,668	1.7	79	12,396
Experimental printer	…	1,632	1,553	0.62	1,441	1.8	0	5,452
Lunch room	…	2,165	1,472	0.67	1,518	2.0	195	62,062
Post-processing	11 to 15 Oct 2021	3,008	1,654	0.78	1,866	2.2	69	425,883
Lunch room	…	1,943	1,524	0.62	1,593	1.9	166	13,333
Ni/Fe printer	…	1,350	1,140	0.52	1,179	1.7	0	8,549
Post-processing	14 to 18 Mar 2022	6,192	2,292	0.85	2,804	2.3	478	645,228
Al printer	…	1,372	1,310	0.40	1,273	1.5	101	6,557
Post-processing	10 to 14 Oct 2022	10,127	1,985	1.1	3,091	2.9	262	3,254,356
Ni/Fe printer	…	1,871	1,742	0.46	1,690	1.6	258	7,896
Ni/Fe printer	16 to 20 Oct 2023	1,059	796	0.59	877	1.8	38	8,554
Depowdering machine	…	3,761	2,717	0.7	2,992	1.9	248	39,208
Band saw	…	1,951	1,301	1.0	1,274	2.6	13	30,021

AM—arithmetic mean; SD—standard deviation; GM—geometric mean; GSD—geometric standard deviation.

The average size of the particles detected by the nano/sub-micron particle measuring devices was 65 nm (SD 23 nm) in diameter for all personal measurements and 73 nm (SD 20 nm) for all stationary measurements (data not in table).

**Figure 1:**
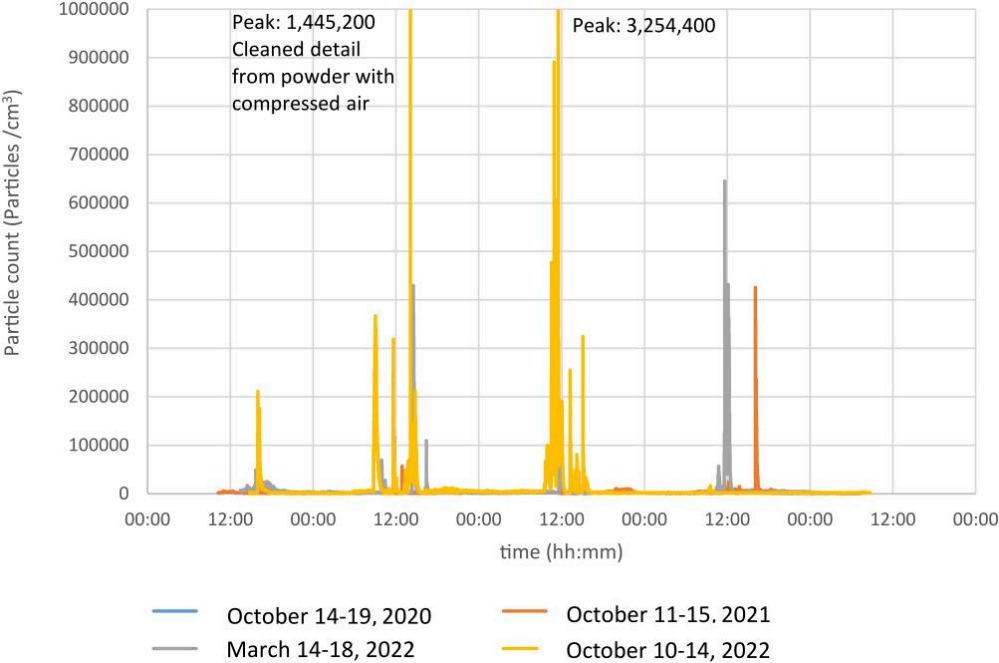
Results from stationary nano/sub-micron particle measurements with NanoTracer/Partector instruments in the additive manufacturing post-processing room at a Swedish AM company during four separate measurement weeks, from Monday afternoon to Friday morning.

**Figure 2:**
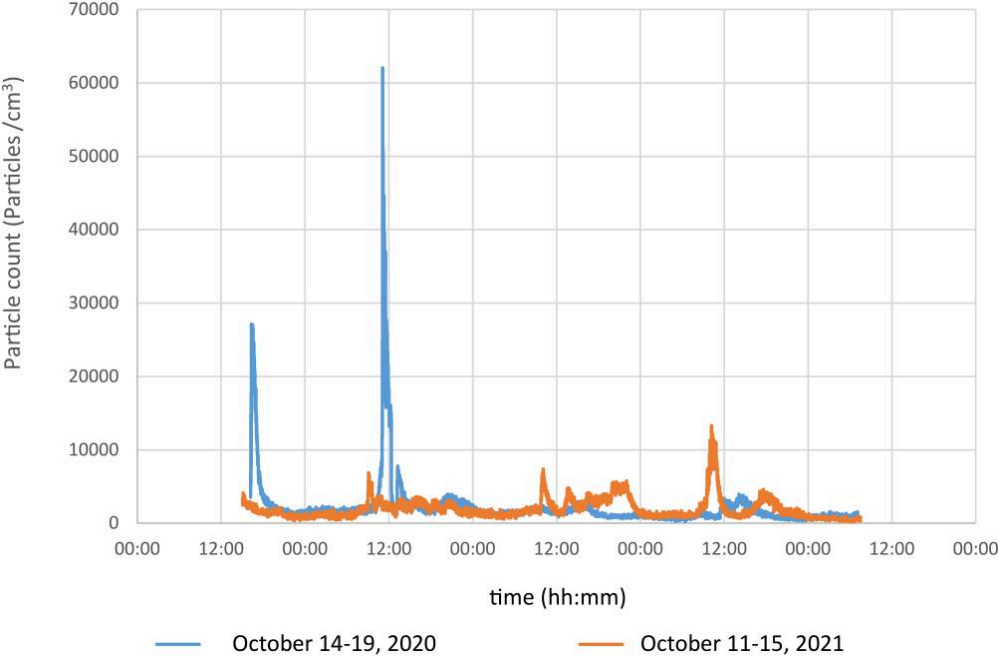
Results from stationary NanoTracer measurements in the lunchroom at a Swedish AM company during two separate measurement weeks. The higher peaks during Monday and Tuesday and lower peaks Wednesday-Friday (October 14 to 19) were due to workers avoiding the lunchroom later in the week because of disturbing noise from the sampling pumps.

The results from the logging nano/sub-micron particle measurements in the post-processing room during four separate measurement weeks, Monday-Friday, are shown in Fig. 1. The results demonstrate several peaks per day, where particle numbers quickly spiked from low baseline levels up to ca 300,000 to 3,000,000 particles/cm^3^ for a short period of time and then quickly returned to baseline. For comparison, the highest peak levels in a busy lunch room reached ca 60,000 particles/cm^3^ (Fig. 2).

### Personal nano/sub-micron particle monitoring across the additive manufacturing facility

Based on the findings from stationary measurements, personal logging instruments were used to assess individual and task-specific particle exposure levels. Nano/sub-micron (10 to 300 nm) particles were measured over the course of a full or partial workday, with 20 personal measurements conducted on workers in production and six on office workers serving as controls. Particle exposures varied between 0 and 2,486,294 particles/cm^3^ (Table 3). The highest number of particles was measured for a worker when dusting off the work clothes with compressed air after work done by the printer on 23 October 2020.

**Table 3:** Number of nano/submicron (10 to 300 nm) particles/cm^3^ by personal sampling with Nanotracer and Partector during the five measurement weeks, Monday-Friday, at a Swedish AM company 2020 to 2023; as presented by workplace.

Work place	Date	N	AM	Median	SD	GM	GSD	Min	Max
						(particles/cm^3^)			
Office	…	6	…	…	…	…	…	…	…
	19 Oct 2020		2,508	1,182	2.3	1,112	9.6	0	26,412
	11 Oct 2021		2,108	1,988	0.40	2,464	1.5	846	4,332
	15 Oct 2021		629	494	2.4	395	11	0	2,385
	15 Oct 2021		629	614	3.8	230	46	0	2,026
	15 Oct 2021		1,510	869	0.80	1,098	2.2	97	5,516
	16 Oct 2023		2,925	1,129	0.97	1,455	2.6	143	34,333
Production		20	…	…	…	…	…	…	…
	19 Oct 2020		2,717	1,041	0.97	1,317	2.6	220	58,385
	19 Oct 2020		7,358	1,676	2.3	1,774	9.9	0	289,080
	23 Oct 2020		7,377	7,168	0.80	5,662	2.2	78	83,984
	23 Oct 2020		10,858	553	3.2	620	25	0	2,486,294
	23 Oct 2020		8,155	767	1.6	1,770	5.2	234	63,115
	11 Oct 2021		4,852	2,128	0.57	2,120	1.8	780	351,814
	11 Oct 2021		2,083	1,786	0.59	1,844	1.8	0	7,563
	15 Oct 2021		2,269	867	2.2	1,028	8.7	0	12,910
	4 Mar 2022		17,872	3,655	1.8	5,857	6.0	0	104,768
	14 Mar 2022		2,140	1,974	0.42	1,962	1.5	61	13,488
	18 Mar 2022		912	814	0.56	789	1.8	98	4,123
	18 Mar 2022		3,640	1,844	0.81	2,067	2.2	549	229,783
	4 May 2022		5,781	2,490	1.2	3,038	3.5	0	148,811
	4 May 2022		1,937	1,040	0.86	1,222	2.4	125	61,601
	10 Oct 2022		23,832	5,786	1.3	9,347	3.8	87	855,004
	14 Oct 2022		1,589	1,305	0.45	1,366	1.6	484	53,304
	16 Oct 2023		1,383	918	0.88	950	2.4	25	13,300
	16 Oct 2023		2,226	1,222	0.98	1,232	2.7	76	188,001
	20 Oct 2023		211	140	0.85	135	2.3	5	10,359
	20 Oct 2023		987	240	1.3	273	3.5	14	48,586

n—number of measurements; AM—arithmetic mean; SD—standard deviation; GM—geometric mean; GSD—geometric standard deviation.

**Figure 3:**
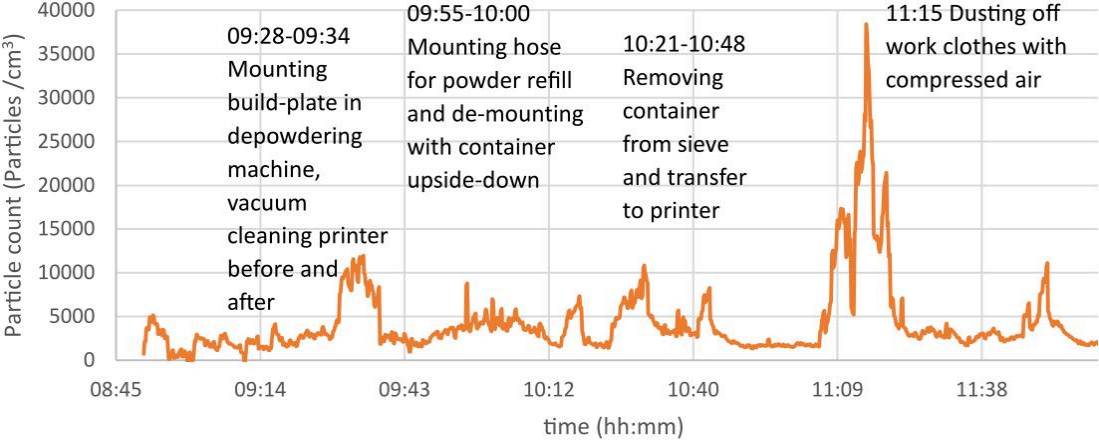
Results from personal NanoTracer measurement at Ti-printer at a Swedish AM company on March 14, 2022.

**Figure 4:**
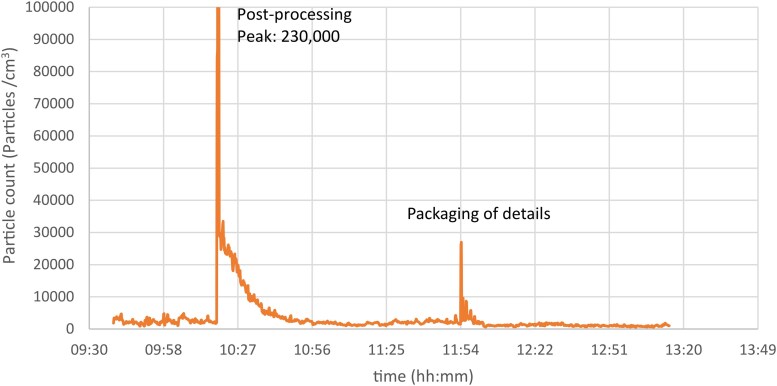
Results from personal NanoTracer measurement at post-processing and packaging of details at a Swedish AM company on March 18, 2022.

Small exposure peaks by personal measurements were identified for AM workers when cleaning the printer (9,100 particles/cm^3^), filling feedstock powder (10,800 particles/cm^3^), and higher exposures when dusting off work clothes by compressed air (38,400 particles/cm^3^), in post-processing (230,000 particles/cm^3^), and packing of printed parts (26,200 particles/cm^3^) (Figs. 3 and 4). Some detailed work-diaries were used to pinpoint the work-tasks associated with time-specific exposure peaks, as demonstrated in Fig. 3, whereas several diaries were not detailed enough to provide reliable information on the work-tasks performed at specific peaks exposures and were thus not included in the data.

### Dust measurements and metal analyses

After quantifying nano/sub-micron particle exposure levels, the dust concentrations were also assessed in order to provide a broader understanding of particulate exposure risks. Low levels of dust were measured across the facility for all dust fractions, and with all measurements displaying dust values below 3% of the Swedish OELs, respectively. Inhalable dust was the only dust fraction that consistently demonstrated dust levels above the laboratory limit of quantification (LoQ) (13/15 samples above) even though dust was collected onto filters during an entire working week, Monday-Friday, instead of a workday that is the procedure usually used for workplace dust measurements. The smaller dust fractions had 33 to 60% of samples above the LoQ. The dust levels, when excluding samples below the LoQ, were as follows: inhalable dust: 0.0061 to 0.14 mg/m^3^, respirable dust: 0.0082 to 0.022 mg/m^3^, total dust: 0.013 to 0.13 mg/m^3^, PM2.5: 0.010 to 0.023 mg/m^3^, and PM10: 0.0089 to 0.079 mg/m^3^ (Table S2). Post-processing generally displayed the highest amount of dust compared to other measured locations and positions—especially for the larger dust fractions that followed the same trends as observed in the nano/sub-micron particle measurements, ie inhalable dust, total dust, and PM10. Dust levels measured in the area by the printers demonstrated levels similar to background levels measured in the lunch room.

For the Sioutas Cascade Impactor, 79% of all samples displayed dust levels below the LoQ, and all quantifiable levels of dust were low (0.0021 to 0.023 mg/m^3^) (Table S3). The majority of the analyzed filters with quantifiable dust levels were measured for particle size <0.25 µm (9/15 filters). In the post-processing area, the highest levels of particle sizes >2.5 µm (0.023 mg/m^3^) and 1.0 to 2.5 µm (0.027 mg/m^3^) were found.

Analyses of metals in the dust fractions revealed low levels in all samples, with all detected metals demonstrating air concentrations below 1% of the Swedish OELs (results not shown). The metal content of dust samples collected at each stationary measurement location is shown in Table 4. The highest metal content was obtained in the post-processing, with samples of inhalable, respirable, and <250 µm dust fractions having at most a metal content between 15% and 20%. The most abundant metals in the samples from the post-processing measurements were iron, aluminum, and chromium. Dust samples collected by the printers had a low metal content, a few percent of the total dust mass, similar to the metal content found in samples from background levels found in the lunch room.

**Table 4:** Metal content of inhalable dust, respirable dust and dust <0.25 µm, from stationary measurements at indicated sampling locations.

	Inhalable dust	Respirable dust	Dust <0.25 µm, sioutas cascade impactor
**Ni/Fe printer (*n* = 3)**			
Dust concentration (range, mg/m^3^)	<0.0047 to 0.026	<0.0070	<0.0023
Detected metals (above LoQ)	**Fe,** Co, Cr, Mo, Ni	…	Mo
Metals, % of total dust mass	0 to 4.8	…	…
**Al printer (*n* = 1)**			
Dust concentration (range, mg/m^3^)	0.012	0.0082	0.0091
Detected metals (above LoQ)	Pb	Pb	Pb, Zn
Metals, % of total dust mass	0.026	0.026	0.071
**Experimental printer (*n* = 1)**			
Dust concentration (range, mg/m^3^)	0.0070	<0.0064	0.0033
Detected metals (above LoQ)	Mo, Ni	Cr, Cu, Fe, Mn, Mo, Ni, Pb, Tl, Zn,	**Ca,** Ba, Cr, Cu, Fe, Mn, Mo, Tl, Zn
Metals, % of total dust mass	0.39	…	3.1
**Post-processing (PP) (*n* = 4)**			
Dust concentration (range, mg/m^3^)	0.0061 to 0.14	<0.0065 to 0.022	<0.0058 to 0.0045
Detected metals (above LoQ)	**Al, Fe,** Ba, Cu, Cr, Co, Mg, Mn, Mo, Ni, Pb, Tl, V, Zn,	**Al, Cr, Fe,** Cu, Mn, Ni, Pb, V, Zn	**Al, Fe,** Cu, Cr, Mn, Mo, Ni, Pb, Tl, V, Zn
Metals, % of total dust mass	0 to 15	4.5 to 17	1.1 to 20
**PP—band saw (*n* = 1)**			
Dust concentration (range, mg/m^3^)	0.0084	<0.0065	0.0021
Detected metals (above LoQ)	**Fe,** Cu	…	…
Metals, % of total dust mass	1.7	…	…
**CNC (*n* = 1)**	0.025	0.016	0.0056
Dust concentration (range, mg/m^3^)	…	…	…
Metals above LoQ	…	…	Mo
Metals, % of total dust mass	…	…	0.08
**PP—Shake out (*n* = 1)**	0.0076	<0.0084	<0.0021
Dust concentration (range, mg/m^3^)	…	…	…
Metals above LoQ	**Fe**	…	…
Metals, % of total dust mass	1.3	…	…
**Lunch room (*n* = 3)**			
Dust concentration (range, mg/m^3^)	<0.0048 to 0.017	<0.0065 to 0.014	0.00040 to 0.0099
Metals above LoQ	Pb, Zn	Cr, Cu, Fe, Mn, Pb, Tl, Zn	**Ca,** Ba, Cu, Fe, Mn, Pb, Sb, Tl, Zn
Metals, % of total dust mass	0 to 0.079	…	0 to 1.8

Dust was collected on each filter over a whole work week (sampling time around 100 hours). The metals analyzed included Al, As, Ba, Be, Ca, Cd, Co, Cr, Cu, Fe, Mg, Mn, Mo, Ni, Pb, Sb, Tl, V and Zn.

n—number of measurements; PP—Post-processing; LoQ—Limit of quantification; Metals highlighted in **bold** letters had a mass concentration >1% in at least one of the dust samples.

## Discussion

The application of metal AM is rapidly expanding, as its advantages are increasingly recognized across a wide range of industries—from biomedicine to aerospace engineering. Given the relative novelty of the method, concerns are emerging regarding the occupational and safety risks associated with exposure during metal AM processes. To date, only a limited number of studies have investigated nano- or sub-micron particle and dust exposure in the metal AM industry, which has been summarized in a recent review by Paulin et al. (2025). Only a handful of these studies have adopted personal particle measurements in the breathing zone (Bau et al. 2020; Jensen et al. 2020; Azzougagh et al. 2021, 2022; Kangas et al. 2023). Most of these studies performed personal particle exposure measurements during certain work tasks, possibly missing important exposure peaks or exposure scenarios. Only one study (Jensen et al. 2020) followed an AM operator for a whole working day, covering pre-printing operations, printing, and post-processing. As such, this study complements previous studies with whole-day AM operator nano/sub-micron particle exposure measurements for multiple operators over an extended period of time.

Nano/sub-micron particle and dust emissions were low in the vicinity of the AM printers, likely due to the use of enclosed printers with separate exhaust and ventilation. This finding aligns with previous studies on dust and particle levels in the AM industry (Oddone et al2021; Assenhöj et al 2023; Kangas et al 2023).

In our study, both respirable and inhalable dust levels were low throughout the facility, often below LoQ, including measurements performed nearby the printers, in the post-processing room, at the depowdering station, and in the labs. In comparison, the largest published study in metal AM companies reported higher levels of inhalable dust at most Swedish AM facilities investigated (Assenhöj et al. 2023), suggesting that the AM facility in our study applies relatively high standards for dust control.

Although dust levels were low or below detection limits using gravimetric methods, transient peaks of high nano/sub-micron particle concentrations were frequently observed in the post-processing room. The highest recorded peak reached more than 3,000,000 particles/cm^3^ which is approximately 30,000 times higher than levels near idle printers (approximately 100 particles/cm^3^) and 50 times higher than those measured in a busy lunch room (60,000 particles/cm^3^). The post-processing included work tasks involving manual cleaning and processing of printed parts, use of machines for depowdering, blasting, heat treatment, and band saw cutting. Our results are in line with previous studies, where the highest peak of nano/sub-micron particle levels was found in the post-processing area (Assenhöj et al. 2023). Interestingly, at the facility of the current study, PPE, including filter-fed respirators, protective overalls, and gloves, was used for work by the printers. However, until 2023, no PPE was used during post-processing tasks, highlighting that exposure risks associated with post-processing easily can be overlooked despite it being a significant site of major nanoparticle emissions. The shift in behavior to implement the use of PPE also during post-processing was a direct outcome of insights gained from our study, as it helped inform both workers and management about a previously unrecognized particle exposure risk. As a result, protective measures were expanded to include post-processing tasks, illustrating the critical role of exposure monitoring in improving workplace safety practices.

Because AM-operators commonly move throughout the facility and perform a variety of tasks during the workday, personal nano/sub-micron particle measurements were conducted alongside stationary measurements to provide a more representative assessment of their actual exposure levels. Personal measurements revealed that three of the AM workers had median exposure levels noticeably higher than those of office workers, indicating that some production staff are exposed to elevated nano/sub-micron particle levels during a large portion of the workday. There is no current OEL for nanoparticles; however, the Institute for Occupational Safety and Health of the German Social Accident Insurance (IFA), has defined a target value of 20,000 particles/cm^3^ above the background exposure level that should not be exceeded over an 8 hour work shift for particles with a density >6,000 kg/m^3^ including eg, metals and metal oxides (IFA 2026). The average nano/sub-micron particle exposures in this study were well below this limit, even for the AM workers with the highest exposures, with the highest average exposure value reaching about 8,000 particles above background.

Personal measurements have the advantage of measuring the exposure in the breathing zone and can often reveal higher exposure levels compared to stationary measurements that are placed further away from the exposure source. However, we did not observe higher peak exposure levels with personal measurements compared to stationary measurements, indicating that the nano/sub-micron particles quickly spread across the room and that stationary sampling has the same capability of detecting exposure levels.

Detailed work diaries enabled the identification of specific work tasks that were either associated with, or independent of, exposure peaks. Notably, several tasks with the potential to generate particles—such as refilling of powder, removing container from sieve and transferring it to the printer, vacuuming printer before and after use, and mounting build plate in the depowdering machine—did not result in significant nano/sub-micron particle release (approximately 10,000 particles/cm^3^ or less). In contrast, the highest exposure levels were observed during post-processing, packaging of printed parts, and when using compressed air, eg, for dusting off clothes. These findings underscore the importance of monitoring emissions not only in expected high-risk areas but also in locations and during tasks not beforehand expected as a source of exposure.

Findings from this and other studies demonstrate that while overall dust and nano/sub-micron particle levels can be kept low in metal AM production facilities, specific work tasks—particularly during post-processing—can still lead to transient peaks in nano/sub-micron particle release, resulting in potential human exposure. Although overall nano/sub-micron particle release levels were generally low compared to other metalworking processes, such as welding (Ljunggren et al. 2019), certain metals commonly used in AM, such as nickel and cobalt, can trigger strong reactions in sensitized individuals, even at low exposures. In addition, the powder materials used in AM printing are constantly under development, which means that exposure profiles may also change over time, potentially leading to particles that are more or less toxic. In this regard, it is important to identify workstations or work tasks with potential particle release, allowing AM companies to evaluate and improve the safety of their workers. This was exemplified in our study, where the highest particle levels were detected in the post-processing room. Based on these findings, the company was advised to require personal protective equipment for tasks performed in this area. Additional recommendations included improving local exhaust ventilation to reduce nanoparticle exposure during post-processing, and to ensure that the ventilation system effectively prevents nanoparticle dispersion to adjacent premises.

The main limitation of this study is that it was conducted at a single AM-facility, which restricts the generalizability of the findings to other settings. As a result, broad conclusions about the dust and nano/sub-micron particle levels in AM facilities should be drawn with caution. Nevertheless, the study provides novel insights into the presence and release patterns of dust and particles in AM operations. Another limitation is the lack of details in several of the work diaries, which limited the ability to identify specific tasks associated with peak exposures. More detailed work documentation could have enhanced the interpretation of exposure patterns. Another limitation is that the measuring devices used in this study detect particles in the size range of 10 to 300 nm, while nanoparticles are defined as particles <100 nm. However, the average size of the measured particles was around 70 nm with a SD of 20 nm, indicating that the large majority of particles had a size of <100 nm. One more limitation is that the SKC Leland Legacy air sampling pump used with the Sioutas Cascade Impactor did not manage to keep the recommended air flow of 9.0 l/min, which might have influenced the size distribution of the collected dust on the filters, with larger particles than intended passing through each step. Another limitation is that MCE filters were used instead of PTFE filters in the Sioutas Cascade Impactor. This can be regarded as another source of distortion of the cutoff points with respect to nominal values because of the so-called “rebound effect”.

## Conclusion

Although respirable and inhalable dust levels in the facility were very low, work tasks generating nano- and sub-micron-sized particle release were identified, particularly during post-processing of printed parts. These findings highlight the importance of acknowledging and monitoring particle exposure risks in AM not only during printing, but also throughout subsequent handling and processing steps. AM companies as well as the end user, need this type of exposure information to provide a safer work environment. Health risks associated with airborne particles are influenced by both exposure levels and the toxicity of the materials involved. Therefore, it is essential to evaluate both the exposure and hazard potential of these materials to more accurately assess occupational and downstream health risks. Only through such a comprehensive assessment can appropriate safety measures, regulatory guidelines, and safer material innovations be developed to ensure a safe and sustainable future for metal AM.

## Supplementary Material

wxag040_Supplementary_Data

## Data Availability

The data underlying this study will be shared on reasonable request to the corresponding author.
