# Supplementary material for: Operation-specific and time-resolved monitoring of occupational nano/sub-micron particle exposure in a Swedish metal additive manufacturing facility
Source: Ann Work Expo Health. 2026 Jun 1;70(4):wxag040. doi: 10.1093/annweh/wxag040 (PMC13223065; doi:10.1093/annweh/wxag040)
Supplement: wxag040_Supplementary_Data [file wxag040_supplementary_data.pdf]

Supplementary material

Operation-specific and time-resolved monitoring of occupational nano/sub-micron particle exposure in a Swedish metal additive manufacturing facility

Lena Andersson[1,2], Andi Alijagic[2,3,4], Anders Johansson[5], Magnus Engwall[4], Eva Särndahl[2,3], Alexander Hedbrant[2,3]

[1] Department of Occupational and Environmental Medicine, Faculty of Medicine and Health, Örebro University, SE-701 82 Örebro, Sweden.
[2] Inflammatory Response and Infection Susceptibility Centre (iRiSC), Faculty of Medicine and Health Örebro University, SE-701 82 Örebro, Sweden.
[3] School of Medical Sciences, Faculty of Medicine and Health, Örebro University, SE-701 82 Örebro, Sweden.
[4] Man-Technology-Environment research centre (MTM), Faculty of Business, Science and Engineering, Örebro University, SE-701 82 Örebro, Sweden.
[5] Department of Occupational and Environmental Medicine, Örebro University Hospital, SE-701 85 Örebro, Sweden.

Supplementary Table S1. Chemical composition of the feedstock powder alloys used

Powder alloy	316L Stainless steel powder	AlSi10Mg Aluminum alloy powder*		Ti6Al4V Titanium based powder	
	Content, wt. % (typical values)	Element	Content, wt. %	Element	Content, wt. %
Fe	Balance	Al	Balance	Ti	Balance
Cr	17.5	Si	9.00-11.0	Al	5.50-6.75
Ni	12	Mg	0.20-0.45	V	3.50-4.50
Mo	2.2	Fe	≤0.55	Fe	≤0.25
Mn	1.5	Mn	≤0.45	O	≤0.17
Si	0.2	Ti	≤0.15	C	≤0.08
		Zn	≤0.10	N	≤0.05
		Ni	≤0.05	H	≤0.012
		Pb	≤0.05	Y	≤0.005
		Sn	≤0.05		
		Cu	≤0.05		

Supplementary Table S2. Concentrations of respirable dust, inhalable dust, total dust, PM 2.5 and PM 10 at measurements in the AM facility during the five measurement weeks 2020-2023

**Measurement place**	**Measurement week**	**Respirable dust** (mg/m³)	**Inhalable dust** (mg/m³)	**Total dust** (mg/m³)	**PM2.5** (mg/m³)	**PM10** (mg/m³)
Post-processing	19-23 October 2020	<0.0065	0.0061	<0.0082	0.014	0.014
Experiment printer		<0.0064	0.0070	<0.0082	0.010	0.011
Lunch room		<0.0065	0.0075	<0.0082	0.010	0.019
Post-processing	11-15 October 2021	<0.0067	0.0067	<0.0083	0.023	<0.0093
Lunch room		<0.0067	<0.0048	<0.0083	<0.0093	<0.0093
Ni/Fe printer		<0.0066	<0.0047	<0.0083	<0.0092	<0.0092
Post-processing	14-18 March 2022	0.018	0.062	0.055	0.014	0.051
Lunch room		0.014	0.017	0.017	0.014	0.029
Al printer		0.0082	0.012	0.013	0.013	0.018
Post-processing	10-14 October 2022	0.022	0.14	0.13	<0.011	0.079
Ni/Fe printer		<0.0066	0.026	<0.0082	<0.0092	<0.0092
CNC machine		0.016	0.025	0.022	0.014	0.021
Ni/Fe printer	16-20 October 2023	<0.0066	0.0094	<0.0082	<0.0094	<0.0094
Depowdering machine		<0.0084	0.0076	<0.0083	<0.0092	0.0089
Band saw		<0.0065	0.0084	<0.0083	<0.0089	<0.0089
**AM**		0.0084	0.023	0.020	0.011	0.019
**Median**		0.0047	0.0084	0.0059	0.010	0.011
**SD**		0.57	1.00	0.94	0.40	0.79
**GM**		0.0070	0.012	0.010	0.0097	0.013
**GSD**		1.8	2.7	2.6	1.5	2.2
**Min**		0.0045	0.0033	0.0058	0.0063	0.0063
**Max**		0.022	0.14	0.13	0.023	0.079
Swedish OEL[1]		2.5	5	-	-	-

< Value below the laboratory detection limit; AM – arithmetic mean; SD – standard deviation; GM – geometric mean; GSD – geometric standard deviation

[1] (SWEA, 2023)

Supplementary Table S3. Levels of dust in the size range <0.25 - >2.5 µm on Sioutas Cascade Impactor filter A-E from measurements at the AM facility during the five measurement weeks 2020-2023

	**Filter**	A	B	C	D	E
	**Size range**	>2.5 µm (mg/m^3)	1.0-2.5 µm (mg/m^3)	0.50-1.0 µm (mg/m^3)	0.25-0.50 µm (mg/m^3)	<0.25 µm (mg/m^3)
**Measurement place**	**Measurement week**					
Post-processing	19-23 October 2020	<0.0020	<0.0020	<0.0020	<0.0020	0.0035
Experiment printer		<0.0019	<0.0019	<0.0019	<0.0019	0.0033
Lunch room		<0.0020	<0.0020	<0.0020	<0.0020	0.0040
Post-processing	11-15 October 2021	< 0.0058	<0.0058	<0.0058	<0.0058	<0.0058
Lunch room		<0.0024	<0.0024	<0.0024	<0.0024	<0.0024
Ni/Fe printer		<0.0043	<0.0043	<0.0043	<0.0043	<0.0043
Post-processing	14-18 March 2022	0.014	0.027	<0.0023	<0.0023	0.0041
Lunch room		<0.0023	<0.0023	<0.0023	<0.0023	0.0099
Al printer		<0.0023	<0.0023	<0.0023	<0.0023	0.0091
Post-processing	10-14 October 2022	0.023	0.012	0.0041	<0.0019	0.0045
Ni/Fe printer		<0.0023	<0.0023	<0.0023	<0.0023	<0.0023
CNC machine		0.0066	0.0032	<0.0021	<0.0021	0.0056
Ni/Fe printer	16-20 October 2023	<0.0023	<0.0023	<0.0023	<0.0023	<0.0023
Shaking machine		<0.0021	<0.0021	<0.0021	<0.0021	<0.0021
Band saw		<0.0019	<0.0019	<0.0019	<0.0019	0.0021
**AM**		0.0044	0.0043	0.0020	0.0018	0.0040
**Median**		0.0016	0.0016	0.0016	0.0016	0.0035
**SD**		0.88	0.86	0.37	0.19	0.60
**GM**		0.0026	0.0025	0.0018	0.0017	0.0034
**GSD**		2.4	2.4	1.4	1.2	1.8
**Min**		0.0013	0.0013	0.0013	0.0013	0.0015
**Max**		0.023	0.027	0.0041	0.0018	0.0099

< Value below the laboratory detection limit; AM – arithmetic mean; SD – standard deviation; GM – geometric mean; GSD – geometric standard deviation

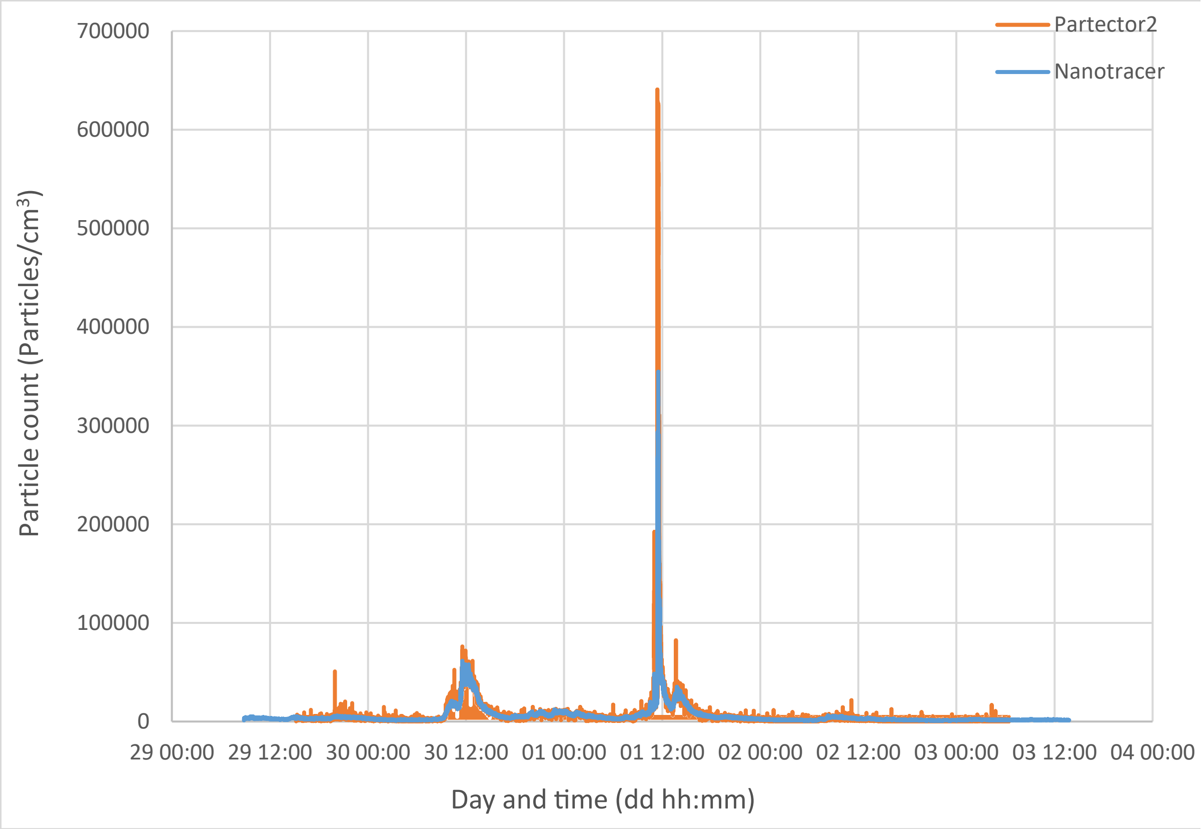

Supplementary Figure S1. Side-by-side comparison of nano/sub-micron particle number concentrations recorded by the Partector 2 and NanoTracer XP instruments. The measurement was performed over a work week in an AM post-processing environment during September 29-October 4, 2025.
